# Pregnancy Outcomes After Maternal Zika Virus Infection During Pregnancy — U.S. Territories, January 1, 2016–April 25, 2017

**DOI:** 10.15585/mmwr.mm6623e1

**Published:** 2017-06-16

**Authors:** Carrie K. Shapiro-Mendoza, Marion E. Rice, Romeo R. Galang, Anna C. Fulton, Kelley VanMaldeghem, Miguel Valencia Prado, Esther Ellis, Magele Scott Anesi, Regina M. Simeone, Emily E. Petersen, Sascha R. Ellington, Abbey M. Jones, Tonya Williams, Sarah Reagan-Steiner, Janice Perez-Padilla, Carmen C. Deseda, Andrew Beron, Aifili John Tufa, Asher Rosinger, Nicole M. Roth, Caitlin Green, Stacey Martin, Camille Delgado Lopez, Leah deWilde, Mary Goodwin, H. Pamela Pagano, Cara T. Mai, Carolyn Gould, Sherif Zaki, Leishla Nieves Ferrer, Michelle S. Davis, Eva Lathrop, Kara Polen, Janet D. Cragan, Megan Reynolds, Kimberly B. Newsome, Mariam Marcano Huertas, Julu Bhatangar, Alma Martinez Quiñones, John F. Nahabedian, Laura Adams, Tyler M. Sharp, W. Thane Hancock, Sonja A. Rasmussen, Cynthia A. Moore, Denise J. Jamieson, Jorge L. Munoz-Jordan, Helentina Garstang, Afeke Kambui, Carolee Masao, Margaret A. Honein, Dana Meaney-Delman, Zika Pregnancy and Infant Registries Working Group, Zika Pregnancy and Infant Registries Working Group, Adriana Rico, Alba Phippard, Alexis B. Peterson, Ana Pomales, Annelise C. Arth, April Dawson, Araceli Rey, Argelia Figueroa, Audilis Sanchez, Brittany Robinson, Daniel B. Williams, Deborah L. Dee, Divia P. Forbes, Elizabeth C. Ailes, Frances Marrero, Gamola Z. Fortenberry, Hilda Razzaghi, Jean Y. Ko, Jennifer N. Lind, Kenneth Lee Dominguez, Kristie Clarke, Maria Flores, Matthew S. Biggerstaff, Melissa Danielson, Monica Molina, Nicholas J. Somerville, Rachel Blumenfeld, Raegan A. Tuff, Rebecca J. Free, Sae-Rom Chae, Sara Andrist, Shin Y. Kim, Tanya L. Williams, Theresa A. Harrington, Tracy Thomason, Vikram Krishnasamy

**Affiliations:** ^1^Division of Reproductive Health, National Center for Chronic Disease Prevention and Health Promotion, CDC; ^2^Division of Congenital and Developmental Disorders, National Center on Birth Defects and Developmental Disabilities, CDC; ^3^Oak Ridge Institute for Science and Education; ^4^Puerto Rico Department of Health; ^5^U.S. Virgin Islands Department of Health; ^6^American Samoa Department of Health; ^7^Division of Human Development and Disability, National Center on Birth Defects and Developmental Disabilities, CDC; ^8^Division of High-Consequence Pathogens and Pathology, National Center for Emerging and Zoonotic Infectious Diseases, CDC; ^9^Division of Vector-Borne Diseases, National Center for Emerging and Zoonotic Infectious Diseases, CDC; ^10^Pacific Island Health Officers Association; ^11^Epidemic Intelligence Service, CDC; ^12^Division of Health Nutrition Examination Surveys, National Center for Health Statistics, CDC; ^13^Division of State and Local Readiness, Office of Public Health Preparedness and Response, CDC; ^14^Office of the Director, National Center for Emerging and Zoonotic Infectious Diseases, CDC; ^15^Division of Public Health Information Dissemination, Center for Surveillance, Epidemiology, and Laboratory Services; ^16^Republic of the Marshall Islands Ministry of Health; ^17^Kosrae Department of Health Services, Federated States of Micronesia.; Division of Emergency Operations, Office of Public Health Preparedness and Response, CDC; Division of Global Migration and Quarantine, National Center for Emerging and Zoonotic Infectious Diseases, CDC; Division of Unintentional Injury Prevention, National Center for Injury Prevention and Control, CDC; Agency for Toxic Substances and Disease Registry; Division of Congenital and Developmental Disorders, National Center on Birth Defects and Developmental Disabilities, CDC; Division of Global Migration and Quarantine; National Center for Emerging and Zoonotic Infectious Diseases, CDC; Division of Congenital and Developmental Disorders, National Center on Birth Defects and Developmental Disabilities, CDC; Division of Global HIV and TB, Center for Global Health, CDC; Division of Reproductive Health, National Center for Chronic Disease Prevention and Health Promotion, CDC; Division of Tuberculosis Elimination, National Center for HIV/AIDS, Viral Hepatitis, STD, and TB Prevention, CDC; Division of Congenital and Developmental Disorders, National Center on Birth Defects and Developmental Disabilities, CDC; Division of Reproductive Health, National Center for Chronic Disease Prevention and Health Promotion, CDC; Epidemic Intelligence Service, CDC; Division of Congenital and Developmental Disorders, National Center on Birth Defects and Developmental Disabilities, CDC; Division of Reproductive Health, National Center for Chronic Disease Prevention and Health Promotion, CDC; Division of Congenital and Developmental Disorders, National Center on Birth Defects and Developmental Disabilities, CDC; Division of HIV/AIDS Prevention, National Center for HIV/AIDS, Viral Hepatitis, STD, and TB Prevention, CDC; Global Immunization Division, Center for Global Health, CDC; Division of Global Migration and Quarantine, National Center for Emerging and Zoonotic Infectious Diseases, CDC; Influenza Division, National Center for Immunization and Respiratory Diseases, CDC; Division of Human Development and Disability, National Center on Birth Defects and Developmental Disabilities, CDC; Division of State and Local Readiness, Office of Public Health Preparedness and Response, CDC; Epidemic Intelligence Service, CDC; Division of Congenital and Developmental Disorders, National Center on Birth Defects and Developmental Disabilities, CDC; Office of the Director, National Center for Chronic Disease Prevention and Health Promotion, CDC; Division of Emergency Operations, Office of Public Health Preparedness and Response, CDC; Division of Foodborne, Waterborne and Environmental Diseases, National Center for Emerging and Zoonotic Infectious Diseases, CDC; Global Immunization Division, Center for Global Health, CDC; Division of Reproductive Health, National Center for Chronic Disease Prevention and Health Promotion, CDC; Division of Reproductive Health, National Center for Chronic Disease Prevention and Health Promotion, CDC; Division of Healthcare Quality Promotion, National Center for Emerging and Zoonotic Infectious Diseases, CDC; Office of the Director, National Center for Chronic Disease Prevention and Health Promotion, CDC; Division of Foodborne, Waterborne and Environmental Diseases, National Center for Emerging and Zoonotic Infectious Diseases, CDC.

Pregnant women living in or traveling to areas with local mosquito-borne Zika virus transmission are at risk for Zika virus infection, which can lead to severe fetal and infant brain abnormalities and microcephaly (*1*). In February 2016, CDC recommended 1) routine testing for Zika virus infection of asymptomatic pregnant women living in areas with ongoing local Zika virus transmission at the first prenatal care visit, 2) retesting during the second trimester for women who initially test negative, and 3) testing of pregnant women with signs or symptoms consistent with Zika virus disease (e.g., fever, rash, arthralgia, or conjunctivitis) at any time during pregnancy (*2*). To collect information about pregnant women with laboratory evidence of recent possible Zika virus infection* and outcomes in their fetuses and infants, CDC established pregnancy and infant registries (*3*). During January 1, 2016–April 25, 2017, U.S. territories^†^ with local transmission of Zika virus reported 2,549 completed pregnancies^§^ (live births and pregnancy losses at any gestational age) with laboratory evidence of recent possible Zika virus infection; 5% of fetuses or infants resulting from these pregnancies had birth defects potentially associated with Zika virus infection^¶^ (*4*,*5*). Among completed pregnancies with positive nucleic acid tests confirming Zika infection identified in the first, second, and third trimesters, the percentage of fetuses or infants with possible Zika-associated birth defects was 8%, 5%, and 4%, respectively. Among liveborn infants, 59% had Zika laboratory testing results reported to the pregnancy and infant registries. Identification and follow-up of infants born to women with laboratory evidence of recent possible Zika virus infection during pregnancy permits timely and appropriate clinical intervention services (*6*).

To characterize pregnancies with laboratory evidence of recent possible Zika virus infection and outcomes of completed pregnancies, data were abstracted from prenatal, delivery, and birth hospitalization records. These abstracted data were included in the Zika pregnancy and infant registries,** which were established by CDC in collaboration with state, territorial, tribal, and local health departments. The number of completed pregnancies with laboratory evidence of recent possible Zika virus infection and a subset with positive nucleic acid tests (NAT)^††^ confirming Zika virus infection (NAT-confirmed) from the registries were analyzed. Pregnancies were included in this analysis if the pregnancy was completed in the U.S. territories on or before April 25, 2017, and reported to the registries on or before May 24, 2017, and if there was laboratory evidence of possible Zika virus infection during pregnancy.

Clinical birth defects experts reviewed abstracted registry data to identify each fetus or infant with birth defects meeting the standard CDC surveillance criteria for possible Zika-associated birth defects (*4*,*5*) and divided them into two mutually exclusive categories: 1) brain abnormalities and/or microcephaly and 2) neural tube defects, eye abnormalities, or consequences of central nervous system dysfunction among fetuses or infants without evidence of other brain abnormalities or microcephaly (*4*,*5*). Analyses were stratified by maternal symptom status^§§^ and trimester of maternal symptom onset or laboratory specimen collection date.^¶¶^ The percentage (with 95% confidence intervals [CI]) of fetuses or infants with possible Zika-associated birth defects was calculated for a binomial proportion using the Wilson score interval.

To describe infant testing and screening (*6*) reported to the Zika pregnancy and infant registries, the percentages of liveborn infants with 1) laboratory testing results for Zika virus infection at birth, 2) postnatal neuroimaging (cranial ultrasound, computed tomography, magnetic resonance imaging, or radiograph) findings, and 3) hearing screening results were calculated. Information about infant testing and screening during birth hospitalization was based on data reported to the registries for births on or before April 25, 2017.

The U.S. territories reported 3,930 pregnancies with laboratory evidence of recent possible Zika infection to the registries during January 1, 2016–May 24, 2017, including 2,549 (65%) pregnancies completed on or before April 25, 2017, which resulted in 2,464 (97%) liveborn infants and 85 (3%) pregnancy losses. Among women with completed pregnancies, 1,561 (61%) reported signs or symptoms compatible with Zika virus infection during pregnancy, 966 (38%) were asymptomatic, and symptom information was missing for 22 (1%). Maternal symptoms or positive laboratory test results were identified in the first, second, and third trimesters for 21%, 43%, and 34% of women, respectively; timing of infection was missing or occurred periconceptionally for 41 pregnancies (2%) (Table 1).

**TABLE 1:** Pregnancy outcomes* for 2,549 completed pregnancies**^†^** with laboratory evidence of recent possible maternal Zika virus infection, by symptom status and timing of symptom onset or specimen collection date — Zika Pregnancy and Infant Registries,^§^ U.S. territories, January 1, 2016–April 25, 2017

Characteristic	No. with brain abnormalities and/or microcephaly^¶^	No. with NTDs and early brain malformations, eye abnormalities, or consequence of CNS dysfunction without brain abnormalities or microcephaly	Total no. with ≥1 birth defect	Total no. of completed pregnancies	Percentage with Zika virus–associated birth defect (95% CI**)
**Any laboratory evidence of recent possible Zika virus infection** ^††^					
Total	108	14	122	2,549	5 (4–6)
**Maternal symptom status** ^§§^					
Symptoms of Zika virus infection reported	68	11	79	1,561	5 (4–6)
No symptoms of Zika virus infection reported	38	3	41	966	4 (3–6)
**Timing**^¶¶^ of symptoms or specimen collection date***					
First trimester^†††^	27	5	32	536	6 (4–8)
Second trimester^§§§^	46	5	51	1,096	5 (4–6)
Third trimester^¶¶¶^	31	4	35	876	4 (3–6)
Recent NAT-confirmed Zika virus infection in maternal, placental, fetal, or infant specimen****					
Total	71	9	80	1,508	5 (4–7)
**Maternal symptom status** ^††††^					
Symptoms of Zika virus infection reported	54	9	63	1,279	5 (4–6)
No symptoms of Zika virus infection reported	16	0	16	225	7 (4–11)
**Timing**^§§§§^ of symptoms or specimen collection date***					
First trimester^†††^	18	4	22	276	8 (5–12)
Second trimester^§§§^	34	2	36	726	5 (4–7)
Third trimester^¶¶¶^	17	3	20	494	4 (3–6)

**Abbreviations:** CI = confidence interval; CNS = central nervous system; IgM = immunoglobulin M; NAT = nucleic acid test; NTD = neural tube defect; RT-PCR = reverse transcription–polymerase chain reaction.

* Outcomes for multiple gestation pregnancies are counted once.

^†^ Includes 2,464 live births and 85 pregnancy losses.

^§^ U.S. Zika Pregnancy Registry and Puerto Rico Zika Active Pregnancy Surveillance System.

^¶^ Microcephaly was defined as head circumference at delivery <3rd percentile for infant sex and gestational age regardless of birthweight. When multiple head circumference measurements were available, the majority of those measurements had to be <3rd percentile for a designation of microcephaly. A clinical diagnosis of microcephaly or mention of microcephaly or small head in the medical record was not required. (https://www.cdc.gov/zika/geo/pregnancy-outcomes.html).

** 95% CI for a binomial proportion using Wilson score interval.

^††^ Includes maternal, placental, fetal, or infant laboratory evidence of recent possible Zika virus infection based on presence of Zika virus RNA by a positive NAT (e.g., RT-PCR), serologic evidence of a recent Zika virus infection, or serologic evidence of a recent unspecified flavivirus infection.

^§§^ Maternal symptom (i.e., fever, rash, arthralgia, or conjunctivitis) status was unknown for 22 completed pregnancies; of these, two resulted in fetuses or infants with brain abnormalities with or without microcephaly.

^¶¶^ Maternal Zika virus infection was reported in the periconceptional period (i.e., the 8 weeks before conception [6 weeks before and 2 weeks after the first day of the last menstrual period]) in 21 completed pregnancies; of these, one resulted in a fetus or infant with brain abnormalities with or without microcephaly. Timing of maternal Zika virus infection was unknown for 20 completed pregnancies; of these, three resulted in fetuses or infants with brain abnormalities with or without microcephaly.

*** Gestational timing of Zika virus infection was calculated using the earliest date of maternal serum, urine, or whole blood collection that tested positive for Zika virus infection by NAT or serologic testing or symptom onset date if symptomatic.

^†††^ First trimester is defined as 2 weeks after last menstrual period to 13 weeks, 6 days gestational age based on estimated date of delivery.

^§§§^ Second trimester is defined as 14 weeks to 27 weeks, 6 days gestational age based on estimated date of delivery.

^¶¶¶^ Third trimester is defined as 28 weeks gestational age or later based on estimated date of delivery.

**** Includes maternal, placental, fetal, or infant laboratory evidence of Zika virus infection based on the presence of Zika virus RNA by a positive NAT (e.g., RT-PCR).

^††††^ Maternal symptom status was unknown for four completed pregnancies; of these, one resulted in a fetus or infant with brain abnormalities with or without microcephaly.

^§§§§^ Maternal Zika virus infection was reported in the periconceptional period (i.e., the 8 weeks before conception [6 weeks before and 2 weeks after the first day of last menstrual period]) in six pregnancies; of these, one resulted in a fetus or infant with brain abnormalities with or without microcephaly. Timing of maternal Zika virus infection was unknown for six pregnancies; of these, two resulted in fetuses or infants with brain abnormalities with or without microcephaly.

Among the 2,549 completed pregnancies, 122 (5%) resulted in a fetus or infant with possible Zika-associated birth defects (5% among symptomatic and 4% among asymptomatic women) (Table 1). The same percentage of birth defects (5%) was observed among the subset of 1,508 (59%) pregnancies with NAT-confirmed Zika virus infections (5% among symptomatic and 7% among asymptomatic women). Among the 122 fetuses or infants that met the surveillance case definition for possible Zika-associated birth defects, 108 (89%) were classified as having brain abnormalities and/or microcephaly. Possible Zika-associated birth defects were reported among pregnant women with symptom onset or positive maternal laboratory test results identified during all trimesters. Among women with symptoms or a positive test result identified during the first, second, and third trimesters, 6%, 5%, and 4% of infants or fetuses, respectively, were reported with possible Zika-associated birth defects. Among pregnancies with NAT-confirmed maternal infections, possible Zika-associated birth defects were reported in 8%, 5%, and 4% of infants or fetuses with maternal symptoms or positive laboratory results identified during the first, second, and third trimesters, respectively.

Among liveborn infants, 59% had Zika laboratory testing results reported to the pregnancy and infant registries. Of the infants, 52% had postnatal neuroimaging findings reported and 79% had hearing screening results reported during birth hospitalization (Table 2).

**TABLE 2:** Infant Zika virus testing and screening at birth for 2,464 live-born infants from completed pregnancies with laboratory evidence of recent possible Zika virus infection — Zika Pregnancy and Infant Registries,* U.S. territories, January 1, 2016–April 25, 2017

Testing and screening	Live-born infants		
	With birth defects^†^ No. (%)	Without birth defects No. (%)	Total No. (%)
**Total**	116 (5)	2,348 (95)	2,464 (100)
**Infant Zika virus testing**			
≥1 infant specimen^§^ test result reported to Zika pregnancy and infant registries	64 (55)	1,381 (59)	1,445 (59)
**Infant screening at birth**			
Postnatal neuroimaging^¶^ conducted and findings reported to Zika pregnancy and infant registries	69 (59)	1,219 (52)	1,288 (52)
Hearing screening conducted and results reported to Zika pregnancy and infant registries	105 (91)	1,840 (78)	1,945 (79)

* U.S. Zika Pregnancy Registry and Puerto Rico Zika Active Pregnancy Surveillance System.

^†^ Includes infants with one or more of the following birth defects potentially associated with Zika virus infection: brain abnormality and/or microcephaly or possible microcephaly, neural tube defect and other early brain malformation, eye abnormality, or consequence of central nervous system dysfunction.

^§^ Infant specimens include serum, urine, and cerebrospinal fluid.

^¶^ Neuroimaging includes any imaging of the infant head, including cranial ultrasound, computed tomography, magnetic resonance imaging, or radiograph reported to the Zika pregnancy registries based on neuroimaging guidance published August 19, 2016. (Russell K, Oliver SE, Lewis L, et al. Update: interim guidance for the evaluation and management of infants with possible congenital Zika virus infection—United States, August 2016. MMWR Morb Mortal Wkly Rep 2016;65:870–8).

## Discussion

Among completed pregnancies with laboratory evidence of recent possible maternal Zika virus infection in the U.S. territories, about one in 20 fetuses or infants had a possible Zika-associated birth defect. When analysis was restricted to NAT-confirmed Zika virus infection in the first trimester, about one in 12 fetuses or infants had a possible Zika-associated birth defect. Zika-associated birth defects were reported after identification of maternal symptoms or positive test results in each trimester.

The overall estimate of 5% of fetuses or infants with possible Zika-associated birth defects among completed pregnancies with NAT-confirmed infections might be affected by the smaller proportion of total completed pregnancies with symptom onset or a positive test result during the first trimester (18%) than during the second or third trimesters (81%). Because available data suggest that the risk for birth defects is higher when infection occurs early in pregnancy (*5,7*) and there are ongoing pregnancies with infection in the first trimester, it will be important to continue to monitor pregnancy outcomes to determine the impact of infection early in pregnancy on the percentage of infants with possible Zika-associated birth defects. Possible Zika-associated birth defects were identified in pregnancies with symptoms or laboratory evidence of recent possible maternal Zika virus infection in each trimester of pregnancy. Challenges with determining the exact timing of infection limit interpretation; however, adverse outcomes following infection throughout pregnancy are consistent with adverse outcomes associated with some other congenital infections (*8*). For example, severe central nervous system sequelae (hearing loss, seizures, or chorioretinitis) have been reported following congenital cytomegalovirus infection later in pregnancy, with the highest risk following first trimester infection (*8*). The continued follow-up of infants is critical to elucidating the impact of Zika virus infection during pregnancy beyond abnormalities detected at birth. Monitoring of ongoing pregnancies with laboratory evidence of possible recent Zika virus infection and the continued follow-up of infant status beyond birth hospitalization can inform public health recommendations for testing, evaluation, and care. Additional information about the full spectrum of outcomes can improve access to early intervention (https://www2.ed.gov/programs/osepeip/index.html) and services for children with special health care needs (https://mchb.hrsa.gov/maternal-child-health-topics/children-and-youth-special-health-needs).

Consistent with previously reported data from the 50 U.S. states regarding primarily travel-associated Zika virus infections in pregnancy, about one in 20 fetuses or infants had possible Zika-associated birth defects (*5*). However, the report from U.S. states included a larger percentage of pregnancies with imprecise timing of infection, thereby limiting any direct comparison of the percentage of affected pregnancies by trimester of infection. This report from the territories, with more robust late pregnancy data, suggests a risk for birth defects throughout pregnancy; further study is needed to confirm this finding. The percentage of infants with possible Zika-associated birth defects after infection identified in the first trimester was 8% (95% CI = 5%–12%) in the U.S. territories compared with 15% (95% CI = 8%–26%) in the U.S. states (*5*); the confidence intervals for these estimates overlap and both are based on relatively small numbers. In addition, for the analysis of the U.S. territories data, a more restrictive definition of confirmed infection, limited to NAT-confirmed infection, was used.

The findings in this report are subject to at least seven limitations. First, the actual number of infants who had Zika virus testing and postnatal screenings might be underestimated because of delays in reporting results to medical records and changes to clinical guidance for infants in August 2016 (*6*). Second, misclassification of microcephaly might have occurred because of imprecise measurements of head circumference at birth and difficulties with consistent surveillance for microcephaly, which could result in overascertainment or underascertainment of microcephaly (*9*). Third, other potential etiologies for these birth defects (e.g., genetic or other infectious causes) were not assessed in this analysis. Fourth, lack of postnatal neuroimaging might have led to underascertaining brain abnormalities; just over half of infants had postnatal neuroimaging reported at birth, despite recommendations that all infants born to mothers with laboratory evidence of possible Zika infection receive such imaging (*6*). Some infants might have additional imaging in the outpatient setting; planned efforts to follow these infants at 2 months and beyond might provide additional data. Fifth, the actual number of Zika virus infections among pregnant women in the U.S. territories might be underestimated. Investigation of a 2007 Zika virus disease outbreak in Yap, Federated States of Micronesia, suggested that up to 80% of Zika virus infections might be asymptomatic or mildly symptomatic (*10*). The percentage of asymptomatic infections in the U.S. territories (38%) was much lower than that reported from Yap and lower than that suggested by data from the Zika pregnancy and infant registries from the U.S. states (62%) (*5*,*10*). However, in the U.S. territories, Zika virus testing of women during pregnancy was recommended regardless of symptom status, whereas a household survey of the general population was conducted in Yap. Sixth, because of limitations in the specificity of current serologic testing, some pregnant women who were reported to the Zika pregnancy and infant registries might have had other flavivirus infections. However, rates of dengue virus transmission were low in Puerto Rico and the U.S. Virgin Islands during 2016 (https://diseasemaps.usgs.gov/mapviewer/), and dengue virus infection is not known to cause birth defects. Finally, some women who were infected with Zika virus before pregnancy might have a persistent immunologic response resulting in a positive immunoglobulin M test detectable during pregnancy. Analyses restricted to pregnancies with NAT-confirmed Zika virus infection indicated a similar proportion of infants with birth defects. However, even with NAT testing, timing of maternal infection might be inexact, especially given that Zika virus RNA might persist during pregnancy (https://www.cdc.gov/zika/laboratories/lab-guidance.html), and because most Zika virus infections are asymptomatic or have mild, nonspecific symptoms.

This report adds information about the number of possible Zika-associated birth defects with laboratory evidence of recent possible or NAT-confirmed Zika virus infection during pregnancy among women living in the U.S. territories and supplements findings from the U.S. states. It also provides new estimates for the proportion of infants with a birth defect after identification of maternal Zika virus infection in the first, second, and third trimesters of pregnancy, and provides evidence that birth defects might occur following documentation of symptom onset or positive laboratory testing during any trimester. Moreover, based on data reported to the pregnancy and infant registries, this report highlights potential gaps in testing and screening of infants with possible congenital Zika virus infection in U.S. territories at birth. Identification and follow-up of infants born to mothers with laboratory evidence of recent possible Zika virus infection during pregnancy can facilitate timely and appropriate clinical intervention services and assessment of future needs (*2,6*). Information about adherence to the recommended newborn testing and screening can improve monitoring and care of infants affected by Zika.

**Summary**

**What is already known about this topic?**

Zika virus infection during pregnancy causes serious brain abnormalities and/or microcephaly and has been associated with other severe birth defects. Local transmission of Zika virus was reported in U.S. territories in 2016.

**What is added by this report?**

Overall, about 5% of fetuses and infants born to women with laboratory evidence of recent possible Zika virus infection in the U.S. territories had possible Zika-associated birth defects, the same as the percentage reported in the 50 U.S. states during 2016. Possible Zika-associated birth defects including brain abnormalities and/or microcephaly were reported following Zika virus infection during every trimester of pregnancy. Among completed pregnancies with positive nucleic acid tests confirming Zika virus infection identified in the first, second, and third trimesters, the percentages of fetuses or infants with possible Zika-associated birth defects was 8%, 5%, and 4%, respectively.

**What are the implications for public health practice?**

Current data suggest that Zika virus infection during any trimester of pregnancy might result in Zika-associated birth defects. Identification and follow-up of infants born to women with laboratory evidence of recent possible Zika virus infection during pregnancy can facilitate timely and appropriate clinical intervention services and assessment of future needs. Information about adherence to the recommended newborn testing and screening can improve monitoring and care of infants affected by Zika.

## Footnotes

* Maternal laboratory evidence of recent possible Zika virus infection was defined as 1) Zika virus infection detected by a Zika virus RNA nucleic acid test (NAT) (e.g., reverse transcription–polymerase chain reaction [RT-PCR]) on any maternal, placental, fetal, or infant specimen (referred to as NAT-confirmed) or 2) detection of recent Zika virus infection or recent unspecified flavivirus infection by serologic tests on a maternal, fetal, or infant specimen (i.e., either positive or equivocal Zika virus immunoglobulin M [IgM] and Zika virus plaque reduction neutralization test [PRNT] titer ≥10, regardless of dengue virus PRNT value; or negative Zika virus IgM, and positive or equivocal dengue virus IgM, and Zika virus PRNT titer ≥10, regardless of dengue virus PRNT titer). Infants with positive or equivocal Zika virus IgM are included, provided a confirmatory PRNT has been performed on a maternal or infant specimen. The use of PRNT for confirmation of Zika virus infection, including in pregnant women and infants, is not routinely recommended in Puerto Rico; dengue virus is endemic and cross-reactivity is likely to occur in most cases (https://www.cdc.gov/zika/laboratories/lab-guidance.html). In Puerto Rico, detection of a positive Zika IgM result in a pregnant woman, fetus or infant (within 48 hours after delivery) was considered sufficient to indicate recent possible Zika virus infection.

† Pregnancies reported to the registries in this report included births or pregnancy losses occurring in the U.S. territories of American Samoa, Puerto Rico, and U.S. Virgin Islands and the U.S. freely associated states of Federated States of Micronesia and Marshall Islands. Outcomes from multiple gestation pregnancies were counted once.

§ Completed pregnancies included live births and pregnancy losses at any gestational age with maternal, placental, fetal, or infant laboratory evidence of recent possible Zika virus infection during pregnancy.

¶ “Birth defects potentially associated with Zika virus infection during pregnancy” refers to the birth defects included in the CDC Zika surveillance case definition (November 2016). The definition covers all birth defects that have been reported as being potentially related to Zika virus infection and includes brain abnormalities, microcephaly (confirmed and possible), neural tube defects and other early brain malformations; eye abnormalities; and consequences of central nervous system dysfunction, such as joint contractures and congenital sensorineural deafness (https://www.cdc.gov/zika/geo/pregnancy-outcomes.html).

** The Zika Pregnancy and Infant Registries include the U.S. Zika Pregnancy Registry (USZPR) and the Puerto Rico Zika Active Pregnancy Surveillance System (PR ZAPSS). The USZPR and PR ZAPSS are both enhanced surveillance systems that collect data on pregnancy and infant outcomes in pregnancies with laboratory evidence of possible Zika virus infection and use similar methods. All U.S. states, the District of Columbia, and all U.S. territories except Puerto Rico are collaborating in the USZPR. Because Puerto Rico has the largest population among U.S. territories, CDC and the Puerto Rico Department of Health established a separate Zika pregnancy registry, called Puerto Rico Zika Active Pregnancy Surveillance System.

†† Pregnancies with nucleic acid tests (NAT) confirming Zika infection include those with a maternal, placental, fetal, or infant specimen in which the presence of Zika virus RNA was documented by a positive NAT.

§§ A pregnant woman is considered symptomatic if one or more signs or symptoms consistent with Zika virus disease (acute onset of fever, rash, arthralgia, or conjunctivitis) is reported. A pregnant woman is considered asymptomatic if these signs or symptoms are not reported.

¶¶ Gestational timing of Zika virus infection was calculated using the earliest date of maternal serum, urine, or whole blood collection that tested positive for Zika virus infection by NAT or serologic testing or symptom onset date if symptomatic. Gestational age dating was based on first trimester ultrasound. If ultrasound was unavailable, dating was based on the last menstrual period. If ultrasound and last menstrual period were unavailable, gestational age was based on information provided on the laboratory requisition form.
